# Impact of PEF and thermal processing on apple juice shelf life

**Published:** 2011-09

**Authors:** AE Torkamani

**Affiliations:** Department of Agriculture and Food systems (DAFS), School of Land and Environment, University of Melbourne, Melbourne, Vic 3010, Australia.

**Keywords:** Pulsed Electric field, High temperature low time, Semirum apple, *Escherichia coli*

## Abstract

**Background and Objectives:**

Pulsed electric field (PEF) is a novel emerging technology which is believed to have the potential to substitute conventional thermal pasteurization (HTST). In the current study PEF was compared with HTST based on microbial inactivation and quality attributes.

**Materials and Methods:**

Juice was prepared by extracting it from Semirum apples. They were chilled to 4°C over night.

Then were divided into two lots, one was treated by PEF and the other by HTST. The treated juices were cultured on tryphtic soy broth (TSB) and results were recorded for 168 days. Quality changes were characterized by color and sensory test. Color changes were quantified using Hunter Lab equipment and equation. Sensory changes were evaluated by test panelists. Results: Using selective media *E. Coli* was enumerated, the total count of the organism was noticeably lower than PEF treated specimen and after 168. The count didn't reach the initial population. Whereas in PEF treated juice bacterial count bounced back to the initial count and exceeds. Results from Hunter Lab indicated a of 3.04 and 3.08 system for PEF and HTST treated juices. Sensory panel showed that PEF is superior to thermal treatment.

**Conclusion:**

The study indicated HTST is more suitable based on food safety encounters. However PEF treated are closer to fresh juices based on quality factors. It can be concluded that PEF has the potential to become a suitable replacement to conventional process if improvements in design are applied.

## INTRODUCTION

With growing consumer interest in healthier and nutritionally rich food, the juice market has had a major growth recently. Although thermal processes cause significant microbial inactivation, many undesired changes have been reported in different studies (1). Enzyme deactivation, color change, alterations in taste and also loss of essential vitamins can be mentioned (1). Due to the mentioned problems caused by conventional thermal processes, more tendencies towards non thermal treatments such as ultrasound, High hydrostatic pressure, irradiation and Pulsed Electric Field (PEF) is witnessed. Among these cold techniques, PEF is growing due to its ability to inactivate organisms and enzymes while no or small temperature elevation is recorded leading to heat sensitive nutrient compound retention (2). Also sensory quality of PEF treated juice is comparable to fresh unprocessed juice (2).

Novel methods such as PEF have been introduced as new processing techniques resulting in improved product quality (3). PEF effect on multiple quality factors in treated products has been studied. Qien et al (4) showed PEF treated apple juice has a shelf life of maximum 3 weeks. Also another study demonstrated PEF capability to decrease microorganisms (3). Regarding to HACCP rules and regulations defined by FDA, fruit juice processors should attain a 5 logarithmical cycle reduction in the most resistant organisms counts by the applied techniques (5). The microbial count should be less than 100 CFU (6).

In this study, we studied the effect of PEF on shelf life in apple juice and compared it with thermal processed juice. For this purpose we considered microorganism enumeration as the main determining factor.

## MATERIALS AND METHODS

Apple juice was extracted using a juice extractor (Mulinex 753 vitafruit) from Semirom apples. Juice was kept at 4C for 24h then filtered. Pasteurization was applied using the high temperature-short time (HTST) method as explained by Moyer and Aitken (7).

Holding temperature was 74.3 for about 25s. The process was done using a container to hold the fluids, coils for juice passage, a pump to circulate juice and thermocouples as a temperature tracker.

The samples were stored in triplicates in pre-sterilized glass bottles (30cc) and evaluated for changes in bacterial count in 14 day periods for 168 days. Bacterial growth count was estimated on tryphtic soy broth (TSB). Incubation was done at 35°C. As *E. coli* had been demonstrated as the main contaminating microorganism in breakouts we considered it as the main pathogen (8). PEF pulses were implemented with bipolar square wave pulses at 0.01-s pulse width, 15 Hz frequency. The device consisted of a probe (P6014A, Tekronix) and a digital oscilloscope (Tekronix). 220 V AC power was converted to 30 kV AC by a transformer and then regenerated to high voltage DC. Flow rate was between 5-50 ml/sec, controlled by a peristaltic pump. The fluid was processed for 1 s. The processed juice was then kept in 3 samples in sterile glass bottles (30cc) and bacterial growth was evaluated as explained before.

**Sensory test**. In order to evaluate the sensory specifica- tion and alteration of the samples, the triangle differenc- es test was performed. A number of panelists, mostly students, were invited and provided with the samples. The juice was settled in opaque glass containers.

**Color**. The color of the treated and control samples was evaluated using hunterlab spectrophotometer (Hunterlab Colorflex, Reston, Virginia, USA). The system iluminant/observer was set at D65/10 which is recommended by the manufacturer and was operated at ambient temperature. The device was calibrated with black and white reference glasses. Samples were transferred to cuvets and data were recorded.

Color difference, ΔE, is calculated by the following equation. (Equation 1)

$$\Delta E=\sqrt{\Delta a^{2}+\Delta b^{\text{2}}+\Delta c^{\text{2}}}$$
(Eq. 1)

Where Δa, Δb and ΔL are difference in red/green, yellow/blue and light/dark ranges respectively. Table 1 explains ΔE based on Cserhalmi et al. work (9). Obtained data were analyzed using SPSS 18software. In order to determine difference between remaining CFU during storage data were rendered by analysis of variance (ANOVA) at significance level of p0.05.

**Table 1:** *index* classification.

Δ*E*	Difference in color
0-0.5	Not noticeable
0.5-1.5	Slightly noticeable
1.5-3.0	Noticeable
3.0-6.0	Well visible
6.0-12.0	Great

## RESULTS

**Fig. 1:**
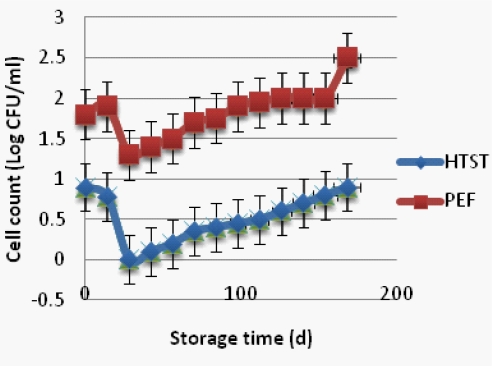
*E.coli* total count during storage.

To evaluate the processing techniques on shelf life, we estimated the bacterial counts in the samples in 14 day intervals. Fig. 1 demonstrates these counts. After analyzing the obtained data a P value of 0.034 was calculated when comparing thermal processing with PEF. Also a mean log count of 0.56 and 1.89 was estimated for thermal and PEF treated juice respectively.

Color test results obtained from Hunter Lab device is represented in Table 1. L is the lightness, a*represents red to green range and b* ranges between yellow and blue (10). L value ranges between 0 and 100 where zero is black and 100 is diffuse white. Negative values for a* represent green while positive indicates red/magnita. Negative range indicates blue color while positive interval determines yellow dominance (10).

We considered a p value less than 0.05 as significant. This was considered as equivalent to about 9 to 15 validated answers out of 18 testers (11). Our results showed that 11 out of 18 testers indicated that the PEF treated juice had better quality attributes than thermally treated samples.

## CONCLUSIONS

As explained, difference in bacterial count between the two methods was meaningful (P value: 0.034) showing that thermal processing is significantly more efficient in bacterial decrease than PEF. It can be depicted from Fig. 1 that bacterial counts decrease in the first 48 days but after that the organisms start the recovery process. Results show zero counts at the 48th day for HTST treated samples. This may be due to the state called “viable but nonculturable cells (VBNC)” where cells do not grow on plates due to metabolical injury (12). In order to detect and count these cells, selective media should be used (12). As TSB is a selective media for enriching fecal coliforms enumeration was possible.

Although the conventional heat processing method was more effective, PEF treated samples constantly had a log count<2.0 that is also acceptable according to marketing standards (6).

Generally when apple juice undergoes Millard brown- ing reactions L value decreases, b* and a* increases (10). This means that the juice becomes darker and more yellow and red. As it is shown in Table 2 the PEF treated juice is lighter, greener and bluer than both fresh and HTST treated juices. Fresh juice has better visual quality attributes than thermally treated juice but less than PEF treated specimens according to Table 2. Calculation quantifies visual significance in juice quality. value for PEF and HTST are calculated as 3.04 and 3.08 which are in the well visible range (Table 1). Triangle test indicated significant difference between PEF and HTST treated juice in taste.

**Table 2:** Comparison of HUNTER Lab variables.

Color Indicators	Fresh	PEF	HTST
L	31	34	28
*a	-1.4	-1.5	-0.9
*b	2.5	2	3

This study showed that, PEF treated juice had no difference in quality from control samples (fresh juice). Our results demonstrated that although HTST is more efficient in microorganism reduction, PEF is an effective and acceptable method, not only suitable for bacterial reduction but also resulting in better flavor and color preservation.
